# Dietary Fiber from Soybean (*Glycine max*) Husk as Fat and Phosphate Replacer in Frankfurter Sausage: Effect on the Nutritional, Physicochemical and Nutraceutical Quality

**DOI:** 10.3390/molecules28134997

**Published:** 2023-06-26

**Authors:** Ana P. Araujo-Chapa, Vania Urías-Orona, Guillermo Niño-Medina, Dolores Muy-Rangel, Ana Laura de la Garza, Heriberto Castro

**Affiliations:** 1 Facultad de Salud Pública y Nutrición, Universidad Autónoma de Nuevo León, Av. Dr. Eduardo Aguirre Pequeño y Yuriria, Monterrey C.P. 64460, Nuevo León, Mexico; 2 Facultad de Agronomía, Universidad Autónoma de Nuevo León, Francisco Villa S/N, General Escobedo C.P. 66050, Nuevo León, Mexico; 3 Centro de Investigación en Alimentación y Desarrollo (CIAD) A.C., Coordinación Culiacán, Carretera Culiacán a El Dorado Km 5.5, Culiacán C.P. 80110, Sinaloa, Mexico

**Keywords:** soybean husk, dietary fiber, phosphate-free, Frankfurter sausage, physicochemical parameters, phenolics, antioxidant capacity

## Abstract

The objective of the present study was to evaluate the effect of dietary fiber from soybean (glycine max) husk as fat and phosphate replacer on the nutritional, physicochemical, and nutraceutical quality of Frankfurter sausage. A traditional formulation was used for the pork-based sausage and three treatments were established: control treatment (CT), sausage without SHDF; treatment 1 (T1), sausage and 1% SHDF; treatment 2 (T2), sausage and 1.5% SHDF. T2 showed the best nutritional contribution of the treatments, significantly favoring a lower content of fat and sodium, thus increasing the contribution of dietary fiber and calcium. A positive effect of SHDF on the water-holding capacity of the treatments was also observed. In addition, T2 remained stable during storage, while T1 and CT showed significantly reduced water-holding capacities of approximately 5%; this was in turn linked to hardness, as it was observed that on day 7 of storage, 27% less force was required to deform the T2 sausages. Regarding color, no significant difference was observed with the addition of SHDF to the product. The results suggest that the dietary fiber extracted from soybean husks has potential for application in food and can be used as an ingredient to improve the functional and nutritional quality of Frankfurter sausages by reducing the content of fat and phosphates.

## 1. Introduction

Meat products provide important components of the diet such as protein of high biological value, fat, minerals, and vitamins with high bioavailability. However, its consumption has been linked to some negative health effects attributed to its components such as lipids, salt, additives, and others, and due to this most consumers consider meat products as less healthy. For this reason, reformulation of meat products has a high development in the last years, exploring different strategies to optimize the composition of these products and make them healthier. There are basically two meat product reformulation strategies: reduce or eliminate certain unhealthy ingredients (e.g., salts, fat) and add healthy ingredients (e.g., natural antioxidants, dietary fiber), or combinations of the two, the latter strategy being the most common. A current trend that is attracting considerable attention is the use of plant-based ingredients as multifunctional compounds in meat products. This is due to the added nutritional value and bioactive compounds and also to technological interest [1].

There is a perception among consumers and often the medical profession that red meat is a food with an excessively high fat concentration. Further, meat fat is considered to cause a variety of human diseases, mainly because of the belief that it has a high proportion of saturated fatty acids (SFA) which raise blood cholesterol levels, a risk factor for cardiovascular disease. Traditional meat products such as sausages are high in fat content which can represent up to 50% of the product formulation [2].

On the other hand, phosphates used in meat processing are the salts of phosphoric acid and sodium or potassium. In the meat industry, phosphates have functions such as buffering capacity, water-binding, emulsification, color stability, oxidation inhibition, antibacterial activity, and protein dispersion properties, but are most commonly used in meat products for their emulsifying and stabilizing capabilities, which largely affect the water-holding capacity [3,4]. Phosphates occur naturally in the form of organic esters in many kinds of food and only 40% to 60% of them are absorbed in the gastrointestinal tract. On the other hand, when phosphates are used as food additives, they are in a free form (not organically bound) and they are actively absorbed with a high potential to generate vascular damage and to induce aging processes. An estimation done in 2012 analyzed the use of phosphates as food additives and concluded that daily intake was around 1000 mg per day and among food with large amounts of phosphates are meat products, including sausages [5].

Dietary fiber is a set of carbohydrates that are not digestible by the human body. It is a component of the cell wall in plant tissues and has multiple benefits if consumed, such as an increase in the intestinal bolus volume, promotion of intestinal transit, prevention of colon cancer, and regulation of blood sugar levels [6]. Dietary fiber can also help to reduce cholesterol levels and has anti-cancer and healing properties [7,8,9]. The incorporation of dietary fiber in food products can also lead to functional benefits because it has a high water retention capacity and can be used as a fat substitute, stabilizer, and texturizer, among others [10].

Dietary fiber is colorless, tasteless, does not affect the sensory quality, and usually also has phenolic compounds associated to it, thus increasing the nutraceutical value of food products [11]. Phenolic compounds are secondary metabolites that are produced in plants during their growth and reproduction, and they have antioxidant properties because they stabilize free radicals [12]. The presence of phenolic compounds in a meat product can play a fundamental role in the stability of fat and proteins [13].

The presence or addition of various plant materials with high contents of phenolic compounds as functional ingredients is increasing in the food industry because of their health benefits. Due to the above-mentioned reasons, continuous work is being conducted regarding the reformulation of sausages to increase their nutritional content without modifying their product characteristics. The addition of plant materials as a functional ingredient with high fiber and phenolic compound contents is an increasingly used technique in the food industry because of the health benefits attributed to them and demands of consumers for healthier products [14,15].

The soybean husk is one of the main by-products derived from soybean oil production and is mainly used as livestock feed [16]. A study conducted previously in our group evaluated the potential as a source of high value-added food products from soybean husk byproduct and the findings showed that this material is composed mainly of dietary fiber but also has high levels of phenolic compounds with antioxidant capacity [17]. In former studies, the phenolic composition of soybean husk has been analyzed, with phenolic acids (ferulic, gallic, vanilic, syringic and chlorogenic) being the most abundant phenolic compounds, and in a minor content has been identified as an isoflavone (Daidzein) [18].

Furthermore, in recent years several works have focused on the use of dietary fiber obtained from different plant materials as fat and phosphate replacers in meat products.

In this regard, several works have reported the partial replacement of fat using dietary fiber. Henning et al. [19] used pineapple dietary fiber as a fat replacer in beef sausage formulation and they reported that the pH, color, and textural properties were similar to the sausage fat treatment. Zhao et al. [20] evaluated the use of regenerated cellulose fiber on the quality and sensory characteristics of fat-reduced emulsified sausages and they concluded that partial replacement of pork back fat with regenerated cellulose fiber could improve the physicochemical and textural property without deteriorating the sensory of emulsified sausage. Souza et al. [21] used inulin, fructooligosaccharide, and α-cyclodextrin as dietary fibers in the formulation of low-fat Italian type salami and concluded that they showed a positive effect on the texture and color parameters in low-fat Italian type salami, and they had good sensory acceptance.

On the other hand, several studies have also evaluated the replacement of phosphates in meat products. In this regard, Powell et al. [22] evaluated the citrus fiber at levels of 0.50%, 0.75%, and 1.00% as a natural replacer of sodium phosphate in bologna sausage and they concluded that citrus fiber replaces some of the functional properties of phosphates and suggested the use of fiber as a contributor in a multi-ingredient phosphate replacement strategy. Later, Câmara et al. [23] used the chia mucilage in powder or gel format as an ingredient for a phosphate replacer in bologna sausage formulations and they found that the use of 2% chia mucilage gel in a phosphate-free and 50% fat reduction sausage showed to be similar in most of the quality attributes with respect to traditional phosphate sausage formulation. At the same time, Magalhães et al. [24] tested the use of bamboo fiber as replacer of phosphate in phosphate-free bologna sausage and they reported that the reformulation of bologna sausages using 5% of bamboo fiber did not change the quality of the final product, and they were sensorially accepted by the consumers.

Therefore, the aim of this work was to evaluate the effect of dietary fiber from soybean husk as fat and phosphate replacers on the nutritional, physicochemical, and nutraceutical quality of Frankfurter sausages.

## 2. Results and Discussion

### 2.1. Characterization of SHDF

#### 2.1.1. Neutral Sugars

The composition of the cell wall differs according to the plant species, stage of development, and environmental conditions. The cell wall mainly comprises polysaccharides including cellulose, hemicellulose, lignin, and pectin, as well as other minor components such as proteins and phenolic compounds that are the components of dietary fiber [25].

Each plant species is characterized by a diversity in the structural composition of its cell walls (which include celluloses, hemicelluloses, and pectin, among others) that endows each plant with certain physicochemical characteristics [26].

The polysaccharides comprising soluble dietary fiber may be different types of sugars such as mannose, galactose, arabinose, and glucose at different concentrations, depending on the source and the method used for its extraction. The content of neutral sugars from SHDF are shown in Table 1. The three main neutral sugars found as components of SHDF were, in order, mannose (≈52%), galactose (≈20%), and arabinose (≈15%), which together comprised 87.89% of SHDF. 

On the other hand, xylose (≈4%), rhamnose (≈3%), glucose (≈3%), and fucose (≈1%) were also found in minor quantities, together representing 12.01% of SHDF. Based on these results, we assumed that the SHDF obtained in the present study was mainly formed by galactomannans, which was in accordance with the description provided by Middelbos and Fahey [27]. 

In addition, our results were similar to those of previously published reports analyzing dietary fiber obtained from soybean husks. In this regard, Shen et al. [28] obtained dietary fiber from black soybean husks using a similar methodology to that of the present work. These authors reported that their dietary fiber mainly comprised mannose (≈62.7%), galactose (≈32.7%), and glucose (≈4.0%), while fucose, xylose, and arabinose were quantified in traces and rhamnose was not included. Han et al. [29] recently extracted soybean husk polysaccharides via hot water extraction (85 °C, 4 h, and pH 4.0) and recovered them as precipitates using ethanol. They also reported that mannose (≈47.5%) and galactose (≈30.4%) were the most abundant monosaccharides, followed by rhamnose (≈7.1%), glucose (≈6.1%), arabinose (≈5.2%), and galacturonic acid, (≈3.4%); xylose was not detected.

Galactomannans are natural and abundant polysaccharides available in legumes with multidimensional aspects which are cost-effective and eco-friendly. The chemical structure is formed by a linear chain of a β-(1–4)-D-mannan backbone with single D-galactose branches attached at an α-(1–6) position and the ratios of galactose and mannose are different. The galactomannans are versatile materials for various applications such as binding, emulsifying, gelling, water retention capacity, suspending, and thickening [30]. Galactomannans can disperse easily in water and form strong hydrogen bonds on interaction with water molecules and, therefore, they have a high water-holding capacity [31].

#### 2.1.2. Mineral Content

Regarding the mineral evaluation of SHDF and considering the total mineral content, Ca represented 47% of total minerals, followed by K (21%) and Na (18%) (Table 2). Guardiola-Márquez et al. [32] mentioned that the negative charges at the surface of dietary fibers allows them to form complexes with minerals due to the cation-exchange capacity with ions such as K^+^, Na^+^, Ca^2+^, and Mg^2+^, which can explain the presence of minerals in SHDF.

In this case, no studies featuring an analysis of mineral content in SHDF could be found, but we noted similarities in mineral content with different plant materials. According to the results reported by Yuliarti et al. [33], calcium was the main mineral comprising soluble dietary fiber obtained from *Cyclea barbata* Myers leaves. A possible explanation for this result is that soluble dietary fibers such as pectin can associate with divalent cations such as calcium due to a high content of negative charges in its carboxyl groups [26].

### 2.2. Characterization of Frankfurter Sausages with Addition of SHDF

#### Nutritional and Mineral Composition

Table 3 shows the results obtained from the analysis of the three Frankfurter sausage treatments on day 0 of storage. A significant difference was observed in moisture content in T1 and T2 compared with CT, indicating that the presence of SHDF in the meat matrix favored water retention in the final product after the cooking process. Dietary fiber is classified into a soluble fraction (hemicelluloses and pectin, among others) and an insoluble fraction (mainly celluloses and lignin), and the soluble fraction could bind water molecules and retain fat in various food products [34].

The results obtained in this study agree with those reported by Vural et al. [35], who worked with 1% dietary fiber from beets, and Choi et al. [36], who worked with 2% dietary fiber from rice bran. Both sets of authors showed that adding dietary fiber to a sausage product favors the water-holding capacity and, therefore, the moisture content. Choi et al. [37] reported up to 20% more moisture in treatments with rice bran fiber and olive oil compared with the control; in our study, T2 (1.5% SHDF) showed a 3% moisture increase. According to Table 3, the protein content did not show significant differences (*p* ≥ 0.05) in T1 and T2 compared with CT, which were similar results to those observed by Ordóñez et al. [38] and Méndez-Zamora et al. [39]. Ash content represents the content of minerals in a product, and T2 was found to be statistically different (*p* ≤ 0.05) compared with CT and T1 in this regard, which was directly related to the addition of SHDF. Campagnol et al. [40] worked with different concentrations of soy fiber in sausages, observing that the higher the fiber content, the higher the ash content. Choi et al. [37] observed a 7% increase in ashes, and we detected an increase of between 5 and 10% in our study.

A current trend in the food industry and research is the formulation and elaboration of healthier products. Particularly in meat products, one strategy is to substitute ingredients that are related to health problems for consumers (cardiovascular problems and obesity, among others), e.g., the fat in sausage products has been replaced by ingredients with greater nutritional and functional contributions [41,42].

In this study, the fat content was reduced by 1% and 1.5% for T1 and T2, respectively, compared with the initial formulation of CT. T1 and T2 presented approximately 1.11% and up to 1.57% less fat, respectively, in accordance with the results reported by Méndez-Zamora et al. [39], who used 1.46% soluble dietary fiber (pectin) and 1.46% inulin as a fat substitute in the formulation of pork sausages; in their study, the fat content of the sausages was reduced by 4.45 to 8.0% compared with the control treatment. Likewise, Yilmaz and Geçgel [43] reported that the use of oat fiber from 3 to 20% in a meatball formulation had a favorable effect in reducing the fat content by 1.1 to 12.8% without affecting the sensory characteristics in the final product.

Each plant source or industrial residue used for the extraction of dietary fiber has significant potential for the formulation of a variety of processed foods, but each dietary fiber will provide different nutritional, functional, and physicochemical characteristics [44].

In this study, following the incorporation of SHDF, T1 and T2 showed greater amounts of total dietary fiber compared with CT, which we expected. Insoluble dietary fiber was the most abundant component in the three treatments but did not show significant differences (*p* ≥ 0.05). Regarding soluble dietary fiber, T1 and T2 showed significant differences (*p* ≤ 0.05) compared with the control; T1 and T2 yielded values three and four-and-a-half times higher than that of CT, respectively. Huang et al. [45] reported a significant increase in the content of dietary fiber in a range from approximately 3 to 6% in Chinese-style sausages by adding 3.5 and 7% of wheat and oat fiber to meat formulations.

Table 4 shows the mineral composition of the Frankfurter sausage treatments. No significant differences were observed in the content of Fe, Mn, Zn, Cu, K, and Mg between the three treatments, but significant differences were observed regarding the contents of Na and Ca. According to the obtained results, there was a significant 18% decrease in the Na content of T2 compared with that of CT. This result may have been related to the water-soluble characteristic of Na, since after the cooking process, it was observed that the sausages treated with SHDF had a higher release of water than control at day 1 of storage.

The Ca results showed increases in T1 and T2 compared with CT because of the addition of SHDF, though T2 showed the higher increase with 80% in the content of calcium compared with CT; these results agree with those reported in Table 2, with Ca being the primary mineral. The dietary fiber of various plant materials has the particularity of interacting with water, thus forming inter- and intra-structural gels. The incorporation of dietary fiber in a food system leads to the formation of a matrix between proteins and minerals such as calcium, which becomes saturated, so excess sodium is lost because it is not able to bind to food. It should be noted that we found no previous studies in relation to the addition of dietary fiber in sausages and its effect on mineral content.

### 2.3. Physicochemical Analysis of Sausages

#### 2.3.1. pH and Titratable Acidity

The pH and titratable acidity of a food are important indicators in the acceptance of the product by consumers, and they also play important roles in shelf life; food products with low pH values avoid microbial growth, thereby increasing their shelf-life [39]. The pH in sausages treated with 1 and 1.5% of SHDF showed significant differences with respect to the control treatment. CT showed a pH value of 6.65, while the pH values were 6.37 and 6.24 in T1 and T2, respectively, so significant differences were observed (Table 5). 

Similar results were observed for sausages treated with chitosan gel at different concentrations, where significantly lower pH values in comparison with control sausages were reported at higher gel concentrations [46]. Álvarez et al. [47] mentioned that some polysaccharides and proteins in food interact through electrostatic associations of attraction and repulsion between polar and nonpolar groups, such that added dietary fiber interacts with meat proteins, decreases the pH, and explains the variations observed in this work. Choi et al. [37] reported that the addition of 2% rice bran fiber and vegetable oil in Frankfurter sausages increased the pH by 3%, reaching values of 6.42, and they attributed this result to the alkaline property of the gum. Therefore, it is important to note that the type of plant material used as an extraction source, as well as the method used to obtain food gums or dietary fiber, significantly affects physicochemical and functional properties of fiber.

The data obtained from the titratable acidity evaluation did not show significant differences between T1 and T2 with respect to CT (Table 5). Herrera-Balandrano et al. [48] reported that sausages treated with 0.3% soluble dietary fiber (arabinoxylan) obtained from nixtamalized maize bran showed increases in titratable acidity compared with a control treatment, which is a different outcome than those observed in the present study.

#### 2.3.2. Weight Loss

In most cases, weight loss has an effect on the quality of food products and is mainly related to water loss. According to Table 6, it was observed that the sausages treated with SHDF did not present a significant difference compared with CT on day 1 of storage with values of 0.33%, 0.55%, and 0.49% in CT, C1, and C2, respectively. However, on the seventh day of storage, CT showed a cumulative weight loss of 2.06% and was significantly different compared with T1 and T2, which showed cumulative weight losses of 0.78% and 0.55%, respectively. The significant differences observed in weight loss in CT respecting to T1 and T2 can be attributed to the functional properties of SHDF.

According to He et al. [49], dietary fiber comprises hydrophilic polysaccharides with high molecular weights, which allows it to retain water between the spaces in the network structured by the fiber when it is used as a food ingredient.

Our results are similar to those reported by Cardoso et al. [50], who observed weight loss of 0.5% and 0.9% at one and twelve days of storage, respectively, in Frankfurter fish sausages formulated with chicory root inulin as dietary fiber. On the other hand, Schmiele et al. [51] reported a weight loss from 1.88% to 5.16% in an emulsified and cooked meat model system formulated with amorphous cellulose as dietary fiber after fourteen days of storage, with these results being different from the present work.

#### 2.3.3. Color

Dietary fiber is composed of tasteless and odorless polysaccharides that do not contribute to color, with a wide range of applications within the food industry, and its effectiveness has been demonstrated in many works [14]. In this study, a color comparison was conducted between day 0 of sausage preparation and day 7 of storage, and significant differences in the values of a* and b* were observed between the treatments, though L* values did not show significant differences, indicating that the addition of SHDF did not affect the luminosity of the product (Table 7).

Significant changes in the color parameters, similar to those found in the present study, were reported by Kim et al. [52] in sausages with 3% pectin added; they similarly obtained lower a* and b* values in treatments with pectin, but they also observed different L* values. Méndez-Zamora et al. [39] did not find significant changes in the color parameters after adding 1.5% pectin and 1.5% inulin to sausages.

#### 2.3.4. Water-Holding Capacity (WHC) and Hardness

T2 showed a water-holding capacity difference of 2.5% from D0 to D7, which was considered relevant in this case since gum retains water for a long time and prevents weight loss (Table 8). Therefore, there were no significant differences between treatments or days of storage. The ability of polysaccharides, dietary fiber, or food gums to interact with water molecules is enabled by their hydrophilic nature [53]. CT, T1, and T2 did not show significant differences at day 0, but T2 showed a significant difference with respect to CT and T1 on the day 7 of storage, which also matches the fact that T2 showed the lower weight loss (0.55%).

Similar results were observed by Méndez-Zamora et al. [39] regarding their treatment with 1.5% pectin and 1.5% inulin. Cengiz and Gokoglu [54] observed an increase in WHC when 5% of citrus fiber was added to pork sausages. On the other hand, according to López-López et al. [33] the water retention of various dietary fibers is related to the type and concentration of their polysaccharides; a high molecular weight and open structure improve hydration properties, as well as water-holding capacity and fat retention. These results explain why the addition of dietary fiber increased the WHC due to the ability of fiber to bind water molecules and retain fat.

Hardness is another parameter that determines the sensory quality of a food. In meat products, fat is one of the main components that contributes to sensory characteristics such as texture [55]. The goal of replacing fat with other food components in the formulation of a food product is to reduce its content without affecting sensory properties. An alternative is the addition of dietary fiber [56].

In Table 8, it can be observed that on day 0 of sausage production, there were no significant differences between treatments, although there were slight decreases in hardness in T1 and T2 compared with CT. On day 7, T2 showed significant 27% and 19% differences compared with CT and T1, respectively, which means that a higher concentration of SHDF in the sausages led to lower hardness values. Individually comparing hardness values of treatments between day 0 and day 7 of storage revealed that T2 did not show a significant difference but CT and T1 did show significant differences with increments of 10.9 N and 11.4 N, respectively. Although we only considered two groups within this study, we note that FDS exerts a protective effect of water retention for longer durations, which also favors less weight loss.

The results obtained in this study agree with those of other reports. Candogan and Kolsarici [57] worked with low-fat Frankfurter sausages with different mixtures of low methoxyl pectin and carrageenan, which decreased the hardness (from 25 to 31%) in formulations compared with the control. In a study carried out by Cierach et al. [55], sausages with carrageenan at different concentrations (0.41%, 0.50%, 0.57%, and 0.70%) improved the hardness of sausages regardless of the amount of carrageenan added; in the case of treatment with 0.70%, hardness increased to 118%, which was opposite to that observed in this study. According to Erçelebi and Ibanoğlu [58], a combination of proteins with polysaccharides, such as dietary fiber or food gums, enables improvements in the emulsifying properties of meat products regarding their physicochemical stability and texture during storage.

### 2.4. Total Polyphenols and Antioxidant Activity

#### 2.4.1. Total Polyphenols

The content of total polyphenols in the different treatments is shown in Table 9, demonstrating a significant increase following the addition of SHDF in the formulation of the meat paste. T1 and T2 showed significantly higher contents of total polyphenols, with increases of 8.55% and 13.48%, respectively, compared with the control treatment. Regarding the analysis during the seven days of storage, no significant differences were observed, so the content of phenolic compounds remained stable. 

The authors of few studies have evaluated the effect of dietary fiber in sausages on the contents of total phenolic compounds. Vivar-Vera et al. [59] evaluated the use of a dietary fiber concentrate obtained from *Averroa carambola* L. in Vienna sausage formulations comprising mixtures of pork and turkey meat. The results reported by the authors match those found in our study, since those authors observed an increase from 69% to 84% in the concentration of total phenolic compounds when adding 2.5% of dietary fiber in different formulations of sausages compared with the control treatment.

On the other hand, Valenzuela-Melendres et al. [60] and Isaza Maya et al. [61] reported on the addition of paste and extracts from different vegetable sources in sausage formulations, showing increases in the content of total polyphenols depending on the composition of the vegetable source and the levels used in the different formulations.

#### 2.4.2. Antioxidant Capacity

Table 10 shows the results of antioxidant capacity of the Frankfurter sausages. Analyzing the DPPH (2,2-diphenyl-1-picrylhydrazyl) antioxidant capacity between the different treatments showed that there was a positive effect of the addition of SHDF. T1 and T2 showed significantly higher antioxidant capacity increases of 66% and 160%, respectively, compared with the control, with T2 being the treatment that showed the highest antioxidant capacity. Regarding the comparison between storage days, no significant differences were observed in antioxidant capacity with respect to storage time, with no changes between D0 and D7.

The addition of SHDF to the formulation of the meat paste led to T1 and T2 showing 13% and 26% increases in ABTS (2,2-azino-bis (3-ethylbenzothiazolin)-6-sulfonic acid) antioxidant capacity, respectively, compared with CT. In the comparison between storage days for each treatment, there was no significant change.

Regarding the FRAP (ferric reducing antioxidant power) antioxidant capacity, no significant difference was found between treatments on day 0; however, the antioxidant capacity increased significantly between treatments on day 7: T2 increased by 51% and T1 increased by 25% compared with the control. Between the storage days, a significant difference was only found in CT, as the antioxidant power decreased by 20%.

Herrera-Balandrano et al. [48] reported that the use of soluble dietary fiber obtained from nixtamalized corn bran at 0.15% and 0.30% in the formulation of Frankfurter sausages increased antioxidant capacity by 30% to 81% according to the DPPH method. However, the authors observed increases from 37% to 81% in the ABTS method, and they observed increases from 102% to 155% in the FRAP method. These results coincide with those of our study, where an increase in the antioxidant capacity was observed regardless of the dietary fiber concentration.

Other authors have reported increases in antioxidant capacity when adding plant-origin ingredients to the formulation of different meat products. Park et al. [62] added an *Achyranthes japonica* extract to pork sausages, and they observed a 34% increase in the antioxidant capacity compared with their control sausage using the DPPH method. Furthermore, Viuda-Martos et al. [63] added orange peel, thyme essential oil, and oregano to the formulation of pork-based sausages, observing a 46% increase in antioxidant capacity using the DPPH method. Evidence of the effectiveness of the addition of some plant materials or extracts in meat products without altering the physicochemical or organoleptic of product characteristics could decrease consumer rejection.

## 3. Materials and Methods

### 3.1. Plant Material

Soybean husks were donated by the Ragasa oil company located in Guadalupe, Nuevo León. All chemical reagents were purchased from Sigma Chemical Co. (St Louis, MO, USA). The raw materials for the sausage (pork meat and fat, garlic powder, ground black pepper, and curing salt) were purchased from a local supermarket.

### 3.2. Dietary Fiber Extraction

The extraction process of dietary fiber from soybean husks was carried out according to the methodology reported by Urias-Orona et al. [64]. The soybean husk was milled to a particle size smaller than 0.5 mm (mesh 35), dispersed in phosphate buffer (100 g/600 mL), and treated enzymatically for starch and protein degradation using α-amylase solution (pH 7, 100 °C, 30 min, 75 U/g sample), amyloglucosidase, (2 h, 60 °C, pH 5.5, 240 U/g sample, 80 rpm), and pronase (pH 7 at 25 °C to 18 h 0.4 U/g sample). Dietary fiber extraction was performed twice under acid conditions, using 0.05 M HCl (1:6 *w/v*) at 80 °C for 1 h at 80 rpm and both supernatants were collected. The extract was centrifuged at 12,040× *g* for 10 min and the pH adjusted to 3.5. The extract was dispersed into 3 volumes of 96% ethanol during 1 h at 4 °C in order to precipitate dietary fiber, which was then collected by filtration through 4 µm Whatman filter paper (Little Chalfont. Buckinghamshire, UK) and on freeze-dried using Labconco FreeZone freez-dryer 6 (Kansas City, MO, USA).

### 3.3. Dietary Fiber Characterization

#### 3.3.1. Neutral Sugars

The content of neutral sugars was determined according to the method of Niño-Medina et al. [65] using alditol acetates. Briefly, dietary fiber samples were hydrolyzed with trifluoroacetic acid (2M) using myo-inositol as the internal standard. Then, samples were converted to alditol acetates using NaBH_4_, followed by acetylation with acetic anhydride using methylimidazole as the catalyst. Finally, samples were injected into an Agilent Varian CP-3800 gas chromatograph using an Agilent DB-23 column at a flow rate of 3 mL/min, an oven temperature of 200 °C, and an injector and detector temperature of 250 °C. Calibration curves were created following the same protocol using rhamnose, fucose, arabinose, xylose, mannose, galactose, and glucose.

#### 3.3.2. Mineral Content

The quantification of minerals was carried out with atomic absorption spectrometry using an Agilent 280 FS system following the methodology of AOAC 968.08 [66]. The samples were ashed at 550 °C and then subjected to acid digestion using HCl; finally, the mineral content was determined with atomic absorption spectrometry using an Agilent 240FS system. The K and Na elements were analyzed via flame emission at 589.6 and 769.9 nm, respectively. In addition, Ca, Mg, Cu, Fe, and Mn were analyzed via absorption at 422.7, 285.2, 213.9, 324.7, 248.3, and 279.5 nm, respectively.

### 3.4. Preparation of Frankfurter Sausages with Addition of SHDF

A formulation was established based on the work of preliminary studies, including those by Herrera-Balandrano et al. [48] and Hur et al. [67], with some modifications and is shown in Table 11. Pork meat was cut into 2 cm pieces and ground for 2 min in a food processor (Robot Coupe, model R2, Jackson, MS, USA); then, we added pork fat and ice (to maintain the temperature at 4 °C), followed by the rest of the ingredients (Table 11). The mixture was kept in the processor for 3 more minutes to achieve complete homogenization and was stuffed into cellulose casings with a diameter of 2 cm and a length of 10 cm. Finally, they were cooked at 80 °C for 40 min in a water bath (Thermo electron 2870, Waltham, MA, USA). Once the process was finished, the samples were immersed in ice water bath at 4 °C for 10 min and then they were packed in resealable bags and stored at 4 °C for 7 days. Three treatments were tested: a control treatment (CT), sausage without SHDF; treatment 1 (T1), sausage and 1% SHDF; and treatment 2 (T2), sausage and 1.5% SHDF.

### 3.5. Chemical Composition of Sausage

#### 3.5.1. Proximate Composition

Composition analysis was carried out according to the established methods of the AOAC [66]: moisture with method 950.46, ashes with method 923.03, fat with method 985.15, protein with the Kjeldahl method (method 992.15), minerals with method 968.08, and total dietary fiber (TDF), soluble dietary fiber (SDF), and insoluble dietary fiber (IDF) with method 985.29.

#### 3.5.2. Mineral Content

The mineral content in the sausage samples was determined as previously described in the dietary fiber section [66].

### 3.6. Physicochemical Analysis of Sausage

Physicochemical parameters were established according to Herrera-Balandrano et al. [48] and Dzudie et al. [68], with minor modifications.

#### 3.6.1. pH

Five grams of sample was weighed and homogenized with 5 mL of distilled water; pH measurements were performed with a pH meter (Corning 440, Woburn, MA, USA), and the analysis was performed in triplicate for each treatment.

#### 3.6.2. Titratable Acidity

To assess titratable acidity, five grams of sausage was taken and homogenized with 100 mL of distilled water; then, the sample was filtered, placed in a 250 mL flask, and topped with distilled water. From this solution, 25 mL was taken and placed in a 150 mL Erlenmeyer flask, and 75 mL of distilled water was added and titrated with 0.01 N NaOH using 5 drops of phenolphthalein as an indicator. The determination was carried out in triplicate, the equation used to determine the % acidity was as follows:

% Acidity = ((V × N × Meq)/M) × 100 × df

where V: volume of NaOH spent; N: normal NaOH; Meq: milliequivalents of lactic acid; M: sample volume; and df: dilution factor.

#### 3.6.3. Weight Loss (WL)

Three pieces of sausage from each treatment were weighed daily during storage. Prior to their measurement, they were kept at room temperature for 30 min, and their weight loss was calculated based on the following formula:

WL (%) = ((Initial Weight − Final Weight)/Initial Weight) × 100.(1)

#### 3.6.4. Color

Samples corresponding to days 0 and 7 of storage were taken for color evaluation using the external–central part of each sausage with a Konica Minolta CR-10 Plus colorimeter based on the CIELAB system (L*, a* and b*); the equipment was calibrated before measurements. All evaluations were carried out in triplicate.

#### 3.6.5. Hardness

To assess hardness, a 40% compression of the mean diameter of the samples was applied using a Chatillon texturometer (model TA1, Scarsdale, NY, USA). The test was carried out using a compression plate with a diameter of 50 mm and a speed of 50 mm/min, with the central part of the sausage used as reference. The analysis was carried out in triplicate for each treatment on days 0 and 7 of storage, and the values are reported in Newtons (N).

#### 3.6.6. Water-Holding Capacity (WHC)

To analyze water-holding capacity, we used a compression method in which we took 0.3 g of a sample and placed it between two pieces of filter paper (Whatman 125 mm); then, the sample was placed between two 20 × 30 cm plastic plates, and a force of 4.0 kg was applied for 20 min. The water impregnated in the paper was considered to be free water. The analysis was performed in triplicate for all treatments on days 0 and 7 of storage.

### 3.7. Nutraceutical Analyses

#### 3.7.1. Total Phenols

The assess total phenol content, extraction was carried out based on the method of Herrera-Balandrano et al. [48] and the extracts were stored at −20 °C until use. The tests were carried out according to the work of López-Contreras et al. [69] and the content of total polyphenols were determined using a Folin–Ciocalteu reagent with gallic acid as the standard (0 to 200 mg/L). The results were expressed as equivalent milligrams of gallic acid by kilogram of sample (mg EAC/kg).

#### 3.7.2. Antioxidant Capacity

Antioxidant capacity was evaluated based on the reduction in the absorbance of radicals 2,2-diphenyl-1-picrylhydrazyl (DPPH) and 2,2-azino-bis (3-ethylbenzothiazolin)-6-sulfonic acid (ABTS) using Trolox as standard (0 to 500 μmol/g), and the results are expressed as equivalent micromoles of Trolox per gram of sample (μmol ET/g). For the ferric reducing antioxidant power (FRAP) assay, a solution of 300 mM C_2_H_3_NaO_2_·3H_2_O was prepared at pH 3.6, with 10 mM of TPTZ (2, 4, 6 tripyridyl-s-triazine) in 40 mM hydrochloric acid and 20 mM of FeCl_3_·6H_2_O at a ratio of 10:1:1. For the test, 0.2 mL of the phenolic extract was mixed with 3.3 mL of the FRAP reagent, using Trolox as a standard, and the reading was performed at 593 nm; the results were expressed as µmol TE/g, following the work of López-Contreras et al. [69] (2015). The analysis was carried out for the three treatments on day 0 (sausages’ preparation) and on day 7 under the corresponding storage conditions.

### 3.8. Statistical Analysis

All of the results were expressed as mean values of three samples ± standard deviation. Statistical significance among samples was evaluated by one-way analysis of variance (ANOVA) followed by Tukey’s test using Minitab 17.0 [70]. A level of probability of *p* < 0.05 (5%) was set as statistical significance.

## 4. Conclusions

The dietary fiber obtained from soybean husks as part of residue from the oil industry has functional potential in the manufacture of sausage meat products. In this study, we evaluated the nutritional contribution and physicochemical properties of soybean husks to Frankfurter sausages without altering their properties, such as color. The Frankfurter sausages with added dietary fiber showed positive quality attributes such as the presence of fiber in the meat product, a lower fat content, and antioxidant properties. Dietary fiber minimized weight loss and hardening in sausages during storage under marketable conditions. Further important results were the observed increase in calcium content and the presence of phenolic compounds, which indicated that the studied dietary fiber could be called an antioxidant fiber; however, further analyses are needed to identify phenolic compounds attached/associated to dietary fiber.

## Figures and Tables

**Table 1:** Neutral sugars content in dietary fiber from soybean husk.

Neutral Sugar	Composition (%)
Manose	52.22 ± 4.70
Galactose	20.37 ± 4.23
Arabinose	15.40 ± 2.11
Xylose	4.44 ± 0.81
Rhamnose	3.39 ± 1.31
Glucose	3.13 ± 0.55
Fucose	1.04 ± 0.26

Values expressed as mean ± standard deviation (n = 3).

**Table 2:** Mineral content in dietary fiber from soybean husk.

Minor Elements (mg/kg)		Major Elements (mg/kg)	
Fe	1353.68 ± 44.41	Na	3183.43 ± 89.67
Mn	18.49 ± 0.29	K	3695.37 ± 23.70
Zn	66.88 ± 1.57	Ca	8435.02 ± 38.00
Cu	8.77 ± 0.11	Mg	935.93 ± 19.48

Values expressed as mean ± standard deviation (n = 3).

**Table 3:** Nutritional composition in Frankfurter sausage at day 0.

Component (%)	Treatment		
	CT	T1	T2
Moisture	64.69 ± 0.34 ^b^	65.79 ± 0.63 ^ab^	66.66 ± 1.03 ^a^
Ash	1.82 ± 0.04 ^b^	1.92 ± 0.07 ^b^	2.17 ± 0.09 ^a^
Protein	15.81 ± 0.30 ^a^	15.56 ± 0.30 ^a^	15.61 ± 0.70 ^a^
Fat	12.85 ± 0.30 ^a^	11.74 ± 0.41 ^b^	11.28 ± 0.45 ^b^
Total carbohydrates	5.11 ± 0.80 ^b^	5.84 ± 1.05 ^a^	5.51 ± 0.90 ^a^
Total dietary fiber	2.51 ± 0.02 ^b^	3.49 ± 0.32 ^a^	3.61 ± 0.34 ^a^
Soluble fiber	0.28 ± 0.05 ^b^	0.85 ± 0.06 ^a^	1.25 ± 0.08 ^a^
Insoluble fiber	2.22 ± 0.21 ^a^	2.64 ± 0.39 ^a^	2.35 ± 0.25 ^a^

CT: Control Treatment, T1: Sausage + 1% SHDF, T2: Sausage + 1.5% SHDF. Values expressed as mean ± standard deviation (n = 3). Different letters within the same row are significantly different (*p* < 0.05).

**Table 4:** Mineral content in Frankfurter sausages on day 0 of storage.

Treatment	Minor Elements (mg/kg)				Major Elements (mg/kg)			
	Fe	Mn	Zn	Cu	Na	K	Ca	Mg
CT	9.50 ± 0.66 ^a^	0.41 ± 0.02 ^a^	4.30 ± 0.22 ^a^	0.44 ± 0.25 ^a^	2207.60 ± 83.10 ^a^	386.40 ± 15.50 ^a^	173.77 ± 1.42 ^b^	55.70 ± 0.05 ^a^
T1	10.73 ± 0.10 ^a^	0.43 ± 0.04 ^a^	4.55 ± 0.16 ^a^	0.24 ± 0.02 ^a^	1690.20 ± 40.8 ^b^	395.37 ± 13.85 ^a^	184.31 ± 0.06 ^a^	52.80 ± 5.26 ^a^
T2	10.01 ± 0.15 ^a^	0.67 ± 0.22 ^a^	4.88 ± 0.06 ^a^	0.28 ± 0.08 ^a^	1484.00 ± 51.40 ^b^	365.32 ± 1.40 ^a^	192.19 ± 3.08 ^a^	54.11 ± 0.26 ^a^

CT: Control Treatment; T1: Sausage + 1% SHDF; T2: Sausage + 1.5% SHDF. Values were expressed as mean ± standard deviation (n = 3). Different letters within the same column are significantly different (*p* < 0.05).

**Table 5:** Physicochemical analysis of Frankfurter sausages on day 0.

Treatments	pH	Titratable Acidity (%)
CT	6.65 ± 0.01 ^a^	0.092 ± 0.01 ^a^
T1	6.37 ± 0.04 ^b^	0.105 ± 0.01 ^a^
T2	6.24 ± 0.02 ^c^	0.108 ± 0.02 ^a^

CT: Control Treatment. T1: Sausage + 1% SHDF. T2: Sausage + 1.5% SHDF. Values expressed as mean ± standard deviation (n = 3). Different letters within the same column are significantly different (*p* < 0.05).

**Table 6:** Weight loss in Frankfurter sausages on days 1 and 7 of storage.

Treatment	Weight Loss (%)	
	Storage Day	
	D1	D7
CT	0.33 ± 0.11 ^a^	2.06 ± 0.48 ^a^
T1	0.55 ± 0.11 ^a^	0.78 ± 0.13 ^b^
T2	0.49 ± 0.49 ^a^	0.55 ± 0.23 ^b^

CT: Control Treatment, T1: Sausage + 1% SHDF, T2: Sausage + 1.5% SHDF. Values expressed as mean ± standard deviation (n = 3). Different letters within the same column are significantly different (*p* < 0.05).

**Table 7:** Color analysis (L*, a*, b*) in Frankfurter sausages on days 0 and 7 of storage.

Storage Day	Parameters	Treatments		
		CT	T1	T2
D0	L*	58.90 ± 0.52 ^a^	58.00 ± 0.50 ^a^	57.70 ± 0.88 ^a^
	a*	15.86 ± 0.20 ^a^	15.13 ± 0.25 ^b^	14.23 ± 0.37 ^c^
	b*	33.03 ± 0.37 ^a^	31.53 ± 0.56 ^b^	29.20 ± 0.43 ^c^
	Color view	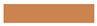	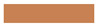	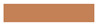
D7	L*	59.10 ± 0.32 ^a^	58.43 ± 0.37 ^a^	58.23 ± 0.46 ^a^
	a*	15.80 ± 0.10 ^a^	14.60 ± 0.43 ^b^	13.46 ± 0.49 ^c^
	b*	31.70 ± 0.47 ^a^	29.36 ± 1.36 ^ab^	27.90 ± 1.17 ^b^
	Color view	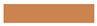	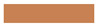	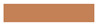

CT: Control Treatment. T1: Sausage + 1% SHDF. T2: Sausage + 1.5% SHDF. Values expressed as mean ± standard deviation (n = 3). Different letters within the same row are significantly different (*p* < 0.05).

**Table 8:** Water-holding capacity and hardness of Frankfurter sausages on days 0 and 7 of storage.

Treatment	WHC (%)		Hardness (N)	
	Storage Day		Storage Day	
	D0	D7	D0	D7
CT	64.21 ± 1.43 ^aA^	59.31 ± 0.08 ^bB^	37.73 ± 2.35 ^aB^	48.66 ± 2.69 ^aA^
T1	65.28 ± 2.97 ^aA^	60.45 ± 1.70 ^bA^	31.99 ± 1.50 ^aB^	43.43 ± 0.14 ^aA^
T2	70.81 ± 1.67 ^aA^	68.31 ± 1.76 ^aA^	29.84 ± 2.87 ^aA^	35.17 ± 1.14 ^bA^

CT: Control Treatment; T1: Sausage + 1% SHDF; T2: Sausage + 1.5% SHDF. Values expressed as mean ± standard deviation (n = 3). Different small letters within the same column are significantly different between treatments (*p* < 0.05). Different capital letters within the same row at same analysis are significantly different between days of storage (*p* < 0.05).

**Table 9:** Total polyphenol content of Frankfurter sausages on days 0 and 7 of storage.

Treatment	Polyphenol Content (mg/kg)	
	Storage Day	
	D0	D7
CT	136.92 ± 5.87 ^bA^	138.46 ± 3.79 ^bA^
T1	148.63 ± 0.14 ^aA^	151.49 ± 2.66 ^aA^
T2	155.38 ± 0.92 ^aA^	160.51 ± 0.25 ^aA^

CT: Control Treatment; T1: Sausage + 1% SHDF; T2: Sausage + 1.5% SHDF. Values expressed as mean ± standard deviation (n = 3). Different small letters within the same column are significantly different between treatments (*p* < 0.05). Different capital letters within the same row are significantly different between days of storage (*p* < 0.05).

**Table 10:** Antioxidant capacity of Frankfurter sausages on days 0 and 7 of storage.

Assay	Treatment	Antioxidant Capacity (µmol Trolox/kg)	
		Storage Day	
		D0	D7
DPPH	CT	73.11 ± 2.55 ^cA^	64.22 ± 2.54 ^cB^
	T1	122.00 ± 9.28 ^bA^	115.88 ± 0.96 ^bA^
	T2	195.33 ± 7.26 ^aA^	192.55 ± 6.73 ^aA^
ABTS	CT	784.22 ± 5.09 ^cA^	785.33 ± 6.66 ^cA^
	T1	893.11 ± 10.18 ^bA^	902.00 ± 8.81 ^bA^
	T2	988.66 ± 13.33 ^aA^	1009.77 ± 6.93 ^aA^
FRAP	CT	313.26 ± 5.29 ^aA^	259.04 ± 18.28 ^cB^
	T1	319.48 ± 19.65 ^aA^	322.15 ± 13.10 ^bA^
	T2	377.93 ± 27.51 ^aA^	392.04 ± 26.34 ^aA^

CT: Control Treatment; T1: Sausage + 1% SHDF; T2: Sausage + 1.5% SHDF. Values expressed as mean ± standard deviation (n = 3). Different small letters within the same column at the same method are significantly different between treatments (*p* < 0.05). Different capital letters within the same row at the same method are significantly different between days of storage (*p* < 0.05).

**Table 11:** Frankfurter sausage formulation by treatment.

Ingredients (%)	Treatments		
	CT	T1	T2
Pork meat	59.7	59.7	59.7
Pork fat	15	14	13.5
Ice	22	22	22
Seasonings *	3	3	3
Curing salt	0.30	0.30	0.30
Dietary fiber	0	1	1.5

CT: Control Treatment; T1: Sausage + 1% SHDF; T2: Sausage + 1.5% SHDF. * Garlic powder, ground black pepper.

## Data Availability

Data are not available.

## Sample Availability

Samples of the compounds are not available from the authors.
